# On the Digital Psychopharmacology of Valproic Acid in Mice

**DOI:** 10.3389/fnins.2020.594612

**Published:** 2020-11-06

**Authors:** John Samuel Bass, Anney H. Tuo, Linh T. Ton, Miranda J. Jankovic, Paarth K. Kapadia, Catharina Schirmer, Vaishnav Krishnan

**Affiliations:** ^1^Department of Neurology, Baylor College of Medicine, Houston, TX, United States; ^2^Departments of Neuroscience, Psychiatry and Behavioral Sciences, Baylor Comprehensive Epilepsy Center, Baylor College of Medicine, Houston, TX, United States

**Keywords:** anticonvulsant tolerability, home-cage monitoring, anticonvulsant side effects, fetal anticonvulsant syndrome, epilepsy psychiatric comorbidities

## Abstract

Antiepileptic drugs (AEDs) require daily ingestion for maximal seizure prophylaxis. Adverse psychiatric consequences of AEDs present as: (i) reversible changes in mood, anxiety, anger and/or irritability that often necessitate drug discontinuation, and (ii) autism and/or cognitive/psychomotor delays following fetal exposure. Technical advances in quantifying naturalistic rodent behaviors may provide sensitive preclinical estimates of AED psychiatric tolerability and neuropsychiatric teratogenicity. In this study, we applied instrumented home-cage monitoring to assess how valproic acid (VPA, dissolved in sweetened drinking water) alters home-cage behavior in adult C57BL/6J mice and in the adult offspring of VPA-exposed breeder pairs. Through a pup open field assay, we also examined how prenatal VPA exposure impacts early spontaneous exploratory behavior. At 500–600 mg/kg/d, chronic VPA produced hyperphagia and increased wheel-running without impacting sleep, activity and measures of risk aversion. When applied to breeder pairs of mice throughout gestation, VPA prolonged the latency to viable litters without affecting litter size. Two-weeks old VPA-exposed pups displayed open field hypoactivity without alterations in thigmotaxis. As adults, prenatal VPA-exposed mice displayed active state fragmentation, hypophagia and increased wheel running, together with subtle alterations in home-cage dyadic behavior. Together, these data illustrate how automated home-cage assessments of spontaneous behavior capture an ethologically centered psychopharmacological profile of enterally administered VPA that is aligned with human clinical experience. By characterizing the effects of pangestational VPA exposure, we discover novel murine expressions of pervasive neurodevelopment. Incorporating such rigorous assessments of psychological tolerability may inform the design of future AEDs with improved neuropsychiatric safety profiles, both for patients and their offspring.

## Introduction

For the foreseeable future, orally administered antiepileptic drugs (AEDs) will remain the first line of defense against seizures in patients with epilepsy. Since AEDs chronically enhance neuronal inhibition and/or diminish excitation without substrate specificity, the high prevalence of anticonvulsant-induced psychiatric and behavioral side effects (PBSEs) should come as no surprise. Such side effects (including alterations in mood, anxiety, irritability, and anger) substantially contribute to disability in epilepsy (Sajobi et al., 2015), and result in suboptimal dosing, poor compliance and/or AED discontinuation in up to 25% of patients (Stephen et al., 2017). Sadly, the highest incidence of PBSEs occur in patients with medically refractory epilepsy and/or pre-existing psychiatric illness (Chen et al., 2017), itself associated with seizure intractability (Hitiris et al., 2007; Krishnan, 2020). These patients remain at risk for PBSE cross-sensitivity (Chen et al., 2018), drastically limiting treatments for a population already vulnerable to suicide and sudden death. The development of AEDs with improved side effect profiles remains a consensus research benchmark (Traynelis et al., 2020). Nevertheless, preclinical assessments of AED “tolerability” in rodents remain largely limited to measures of acute motor “toxicity” (Wilcox et al., 2020) (e.g., rotarod or open field testing) and/or subjectively scored ordinal scales of well-being (e.g., Irwin screen), which may not capture changes in anxiety, mood, motivation or sociability.

Fortunately, AED-induced PBSEs typically resolve with drug discontinuation (Kanner, 2016; Tolchin et al., 2020). However, in pregnant women with epilepsy, AED exposure during fetal neurodevelopment is associated with an increased risk for anatomic and pervasive cognitive/emotional teratogenic effects. Structural malformations (Tomson et al., 2018), ranging from polydactyly to neural tube defects, may require surgical correction for medical or cosmetic purposes and can be recognized early (including on prenatal ultrasounds), allowing for early surgical intervention. In contrast, autism, attention deficit and intellectual disability are typically recognized in infancy to early childhood (Velez-Ruiz and Meador, 2015; Veroniki et al., 2017), beyond a theoretical critical period during which medical/behavioral interventions may be preventative. Since clinical trials for new AEDs exclude pregnancy, and since randomized blinded controlled trials of AED safety in pregnancy are unethical, our knowledge of AED teratogenicity has come almost exclusively from pregnancy registries and prospective cohort studies (Bromley et al., 2014; Weston et al., 2016). Broadly, these results confirm that teratogenic side effects are drug-specific and occasionally dose-dependent (Meador et al., 2013). Valproic acid (VPA), used as monotherapy or polytherapy is associated with the highest teratogenic risks across both structural and cognitive domains (Harden et al., 2009; Wood et al., 2015; Cohen et al., 2019). With increased awareness and prescriber education, there have been declines in VPA use during pregnancy and associated declines in malformation rates (Tomson et al., 2019). However, consensus recommendations regarding the relatively improved neuropsychiatric teratogenic safety profile (*or lack thereof*) for many newer anticonvulsants are conspicuously absent (Harden et al., 2009; Bromley and Baker, 2017). In support of a direct teratogenic role for valproate, offspring born to pregnant rodent dams (rats or mice) challenged briefly with valproic acid at the approximate time of neural tube closure display behavioral phenotypes deemed “autism-like,” and which are also observed in several genetically informed mouse models of autism (Crawley, 2007; Kazdoba et al., 2016; Tartaglione et al., 2019; Chaliha et al., 2020). While these studies have unraveled some mechanistically informative insights (Roullet et al., 2013; Nicolini and Fahnestock, 2018; Tartaglione et al., 2019; Chaliha et al., 2020), two main caveats limit their translational potential. First, anticonvulsant exposure in pregnant women with epilepsy involves dose-adjusted intake for the entirety of gestation. Second, abnormalities in “sociability,” repetitive and exploratory behavior are typically deduced from brief “out-of-cage” task-based assays (e.g., three-chamber sociability, open field testing, etc.), which are particularly vulnerable to the confounds of human presence and bias (Spruijt et al., 2014; Jankovic et al., 2019).

In this study, we apply instrumented home-cage monitoring (Loos et al., 201 015; Robinson et al., 2018; Jankovic et al., 2019) to non-invasively and unobtrusively assess the psychopharmacology of adult and prenatal VPA exposure in C57BL/6J mice. As opposed to VPA injections applied during specific gestational windows (Roullet et al., 2013; Nicolini and Fahnestock, 2018) or administered *acutely* to demonstrate *acute* seizure protection (Nau and Loscher, 1982; Watanabe et al., 2010), we apply VPA enterally, dissolved in drinking water (Loscher and Nau, 1982; Frisch et al., 2009; Smeland et al., 2012; De Caro et al., 2019; Citraro et al., 2020). By combining undisturbed recordings of spontaneous behavior with carefully selected provocative maneuvers, we illustrate an ethologically sound approach to ascertain drug-induced changes in specific behavioral domains (e.g., feeding, sheltering, etc.) as well as higher-order features of murine behavioral organization.

## Materials and Methods

### Mice and Drugs

Experimental protocols were approved by the Baylor College of Medicine Institutional Animal Care and Use Committee (IACUC). All studies were conducted in accordance with United States Public Health Service’s Policy on Humane Care and Use of Laboratory Animals. Male and female C57BL/6J mice (#000664, Jackson Laboratories) were bred and weaned at 21 days of age within our vivarium, set to a 12 h light cycle (lights ON from 0500 to1,700) under controlled temperature (20–26°C) and humidity (40–70%) settings. Water and chow (PicoLab Select Rodent 5V5R Standard Diet, with 3.6 ppm of folic acid) were provided *ad libitum*. Cages were furnished with cellulose bedding (Biofresh). For VPA dosing, we differentiated between *intended* doses and *actual doses* (determined by bottle and body weight measurements). In pilot experiments with 6–10-weeks-old C57BL/6J mice, actual doses of ∼600 mg/kg/d were obtained by dissolving 2.6 mg/ml of sodium valproate (Sigma Aldrich) in drinking water (sweetened with 0.8% sucrose). Pentylenetetrazole (PTZ) was dissolved in sterile-filtered normal saline and injected intraperitoneally (30 mg/kg, 5 ml/kg). To model fetal exposure, breeder pairs were assembled and randomized to receive similar VPA (*n* = 12) or control solutions (*n* = 10) (Frisch et al., 2009). To avoid unnecessary environmental stressors that may compromise successful breeding and early parenting activities, every attempt was made to limit the physical manipulation of breeder/pup units. Bottles were replaced weekly and switched to standard drinking water at parturition, determined by daily external cage checks. At the end of experiments, all mice were euthanized by CO_2_ inhalation.

### Home-Cage Monitoring and Video-Tracking

Remote behavioral telemetry was conducted as previously described (Jankovic et al., 2019) within a satellite study area termed the BMU (behavior monitoring unit) replicating vivarium lighting, light cycle, humidity and temperature settings. Mice were housed within one of sixteen Phenotyper (Noldus Information Technology) home-cages (30 × 30 × 47 cm) with clear plastic walls, one or two water sources fitted with lickometers (detecting capacitance changes), a feeding meter (measuring beam breaks) and a detachable running wheel (utilizing a wheel-attached magnet and a wall-attached magnet sensor). Bedding and chow were identical to those provided in vivarium cages. A sound machine (“Lectrofan, Adaptive Sound Technologies”) played continuous white noise. Aerial video recordings were conducted using a ceiling-mounted infrared camera together with an array of ceiling mounted infrared lamps. An infrared-lucent shelter (10 × 10 × 5–6 cm) was deployed for all non-dyad studies. The “shelter zone” was defined on a distance-calibrated arena for each cage. Recordings were initiated at a specific clock time or started manually (Figures 2K, 4B, 5B). Live center-point tracking was conducted via Ethovision XT 14 (Noldus) using dynamic subtraction at a sample rate of 15/s. Licking, feeding and wheel-running data were integrated through a hardware control module. For light spot testing (Aarts et al., 2015; Jankovic et al., 2019) (between 1,900 and 2,000), a ceiling-mounted white LED produced a gradient of light, measuring ∼670 lux at the left upper quadrant and ∼27 lux at the right lower quadrant of the cage. A 60 s long 2,300 Hz “beep” was then administered at 2,100. To avoid phenotypic drifts secondary to the experience of long-term voluntary exercise (Guo et al., 2020), running wheels were affixed and subsequently detached within 20–23 h. Convulsive seizures were detected by integrating video and real-time changes in mobility, as described previously (Jankovic et al., 2019). Dyads (in Figure 5) were assembled as sex- and condition-matched pairs of singly housed mice (depicted in Figure 4). Since subjects were unmarked, tracking results are presented as “sum distances” and distance between subjects (DBS). Satellite access was restricted to CS, JSB, and VK who wore a gown, cap, face mask and gloves at all times, and entered at least once daily to visually inspect food and water sources, assess general mouse wellbeing and/or administer injections. Satellite veterinary inspections were performed every other week (∼1,100–1,200).

### Pup Open Field

Pup video recordings were conducted within a square open field made of Lightaling building bricks (Amazon) with a side 14.5 and 7.7 cm high walls. This brick frame was positioned over an absorbent bench pad which was warmed over a circulating water blanket and replaced after every set of mice studied. A KF-TM2515T tripod (K&F Concept) combined with a Kimire Digital Camcorder (1080P, 24 megapixels) was used to obtain 15 min long aerial open field recordings. Ethovision XT was configured to identify time in “center” (a concentric square of 7.25 cm side) and entries into each of four “corners” (3.6 × 3.6 cm). Serial measurements of open field behavior (without drug exposure, Figure 3D) were performed on a large cohort of C57BL/6J pups, for which mice were subjected to no more than three evaluations separated by at least 3–4 days. In contrast, for Figure 3E, all pups were studied *serially* at P5, P10, and P15 (post-natal day 15).

### Data Analysis

**FIGURE 1:**
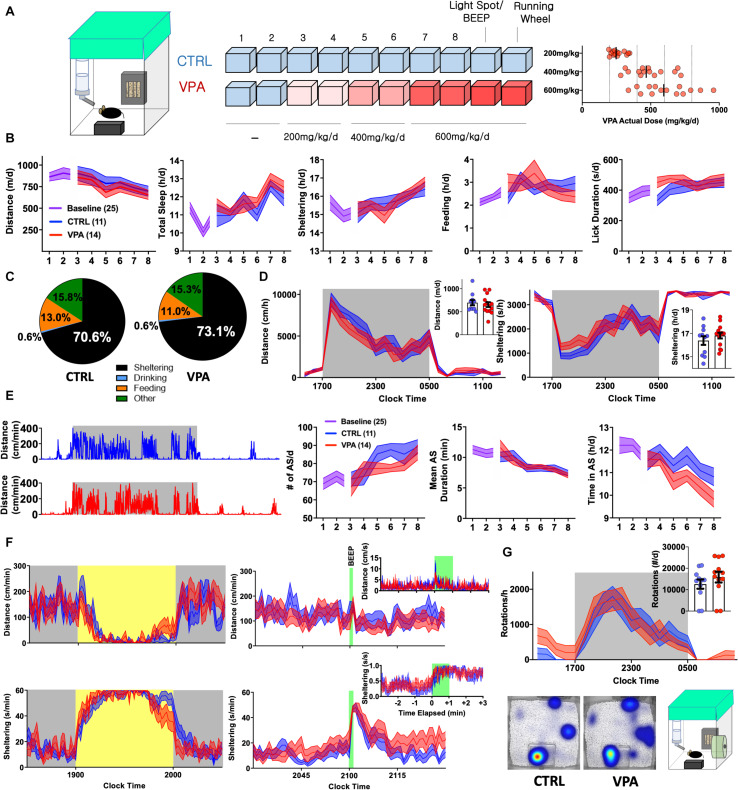
Early Behavioral Consequences of VPA Exposure [CTRL *n* = 11 (5 female) VPA *n* = 14 (7 female)]. **(A)** Schematic of 10-day home-cage protocol, during which VPA is increased to an intended daily dose of 600 mg/kg/d. RIGHT: Actual consumed daily doses (derived from bottle and body weights). **(B)** Home-cage behavioral parameters represented as daily totals (see Supplementary Table S1 for repeated measures analyses). **(C)** Time budgets, and **(D)** hourly/total distances and sheltering parameters measured on day 8. **(E)** Representative actograms (day 8) and quantification of active state parameters. **(F)** Effects of light spot (left) and “beep” testing (right) on measures of distance and sheltering (day 9). **(G)** Wheel-running (day 10) with representative heat maps of position probability. Mean ± SEM shown for all. CTRL, control; VPA, valproic acid; d, day.

Graphs were plotted and analyzed with Prism GraphPad 8 and Microsoft Excel 2016 (Supplementary Figures S2C,D). Time budgets (e.g., Figure 1C) were calculated by tallying total *durations* of sheltering, feeding and drinking. “Sleep” epochs were computed as at least 40 s long contiguous time periods devoid of “movement” (defined as sample velocity ≥ 1.2 cm/s), an approach that has been previously validated electroencephalographically (Pack et al., 2007) and pharmacologically (Jankovic et al., 2019). Active states were defined as epochs lasting at least 1 min long during which mice consistently traversed at least 5 cm/min (see Supplementary Figure S2 for illustration). For dyads, social bouts were defined as at least 3 s long epochs during which DBS consistently measured ≤ 4 cm. Unpaired two-tailed Student’s *t*-tests were used to compare two group means. Repeated measures analysis of variance (RMANOVA) was conducted by fitting a mixed effects model, with main effects and interactions listed in Supplementary Table S1. Two-tailed Chi squared analyses were applied to compare categorical variables across two groups.

**FIGURE 2:**
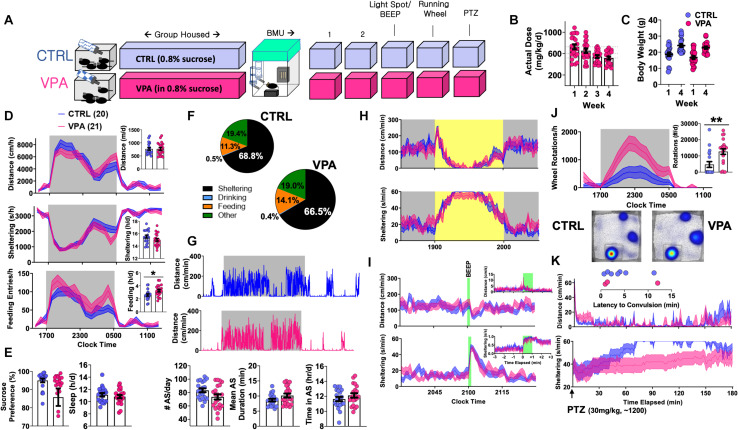
Behavioral Consequences of Chronic VPA Exposure [CTRL *n* = 20 (11 female), VPA *n* = 21 (11 female)]. **(A)** Schematic of protocol, with 4 weeks of VPA exposure at an intended daily dose of 600 mg/kg/d, followed by 5 days of BMU recordings. **(B)** Estimates of actual consumed daily doses. **(C)** Weight change over 4-week treatment period. **(D)** On day 2, total and hourly measures of distance, sheltering and feeding, presented as both feeding duration and feeding entries. **(E)** Sucrose preference, behaviorally defined “sleep” and **(F)** time budgets all obtained from day 2. **(G)** Representative actograms and quantification of active state parameters (day 2). **(H)** Effects of light spot and **(I)** “beep” testing on measures of distance and sheltering. **(J)** Wheel-running behavior with representative heat maps of position probability. **(K)** Changes in distance and sheltering following a single 30 mg/kg intraperitoneal PTZ injection, with inset depicting latency to convulsive seizures. Mean ± SEM shown for all. CTRL, control; VPA, valproic acid. ^∗^*p* < 0.05, ^∗∗^*p* < 0.01.

**FIGURE 3:**
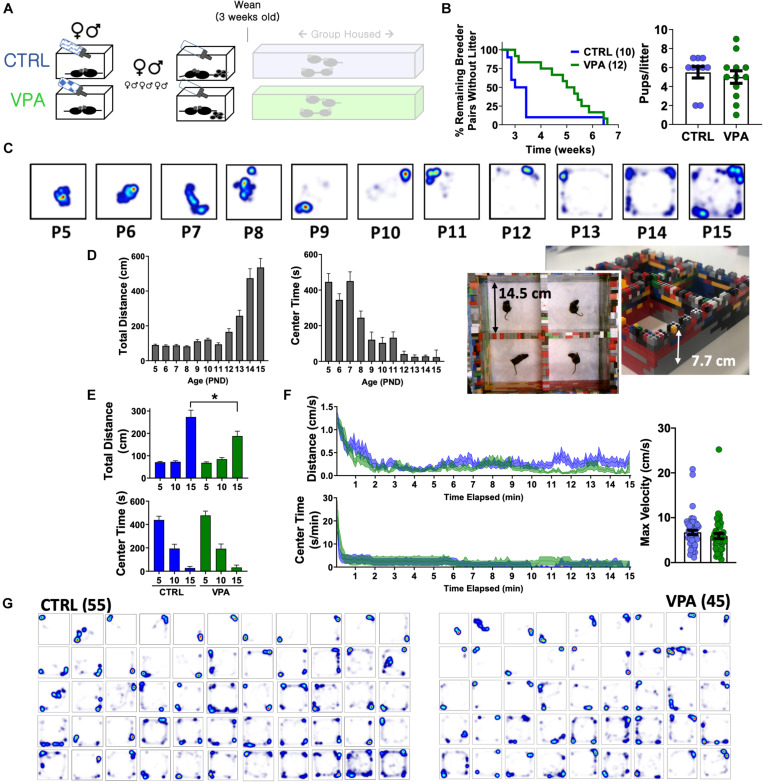
Pup open field behavior following prenatal VPA exposure. **(A)** Breeder pairs were exposed to VPA or control solutions until parturition. **(B)** VPA-exposed breeder pairs took longer to produce a viable litter (*p* < 0.05, Mantel-Cox log-rank test), without a change in litter size. **(C)** Representative heat maps of position probability from a separate cohort of C57BL/6J mice (*n* = 27—45/group), illustrating progressive changes in open field exploration. **(D)** Distances and center times for C57BL/6J mice. **(E)** Total distances and center times across CTRL and VPA cohorts, and **(F)** time-dependent changes in behavior at P15. **(G)** Heat maps of position probability for all pups studied at P15 ranked by overall cage exploration (CTRL *n* = 55 (32 female), VPA *n* = 45 (21 female)]. Mean + SEM shown for all. CTRL, control; VPA, valproic acid. P15 = post-natal day 15. ^∗^*p* < 0.05.

**FIGURE 4:**
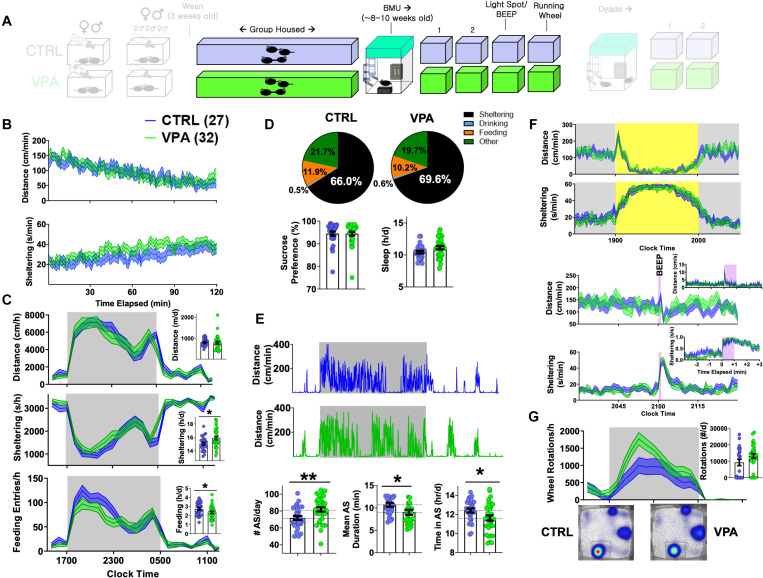
Adult home-cage behavioral profile following prenatal VPA exposure. CTRL *n* = 27 (14 female), VPA *n* = 32 (14 female). **(A)** After weaning, mice were group housed until 8–9 weeks of age. **(B)** Dynamic changes in locomotor activity and sheltering behavior in the first 2 h of BMU recording. **(C)** Hourly/total measures of distance, sheltering and feeding (day 2), and **(D)** corresponding time budgets, sucrose preference and measures of “sleep.” **(E)** Representative actograms and quantification of active state parameters. **(F)** Effects of light spot and “beep” testing on measures of distance and sheltering. **(G)** Wheel-running behavior with representative heat maps of position probability. Mean ± SEM shown for all. CTRL, control; VPA, valproic acid. ^∗^*p* < 0.05, ^∗∗^*p* < 0.01.

**FIGURE 5:**
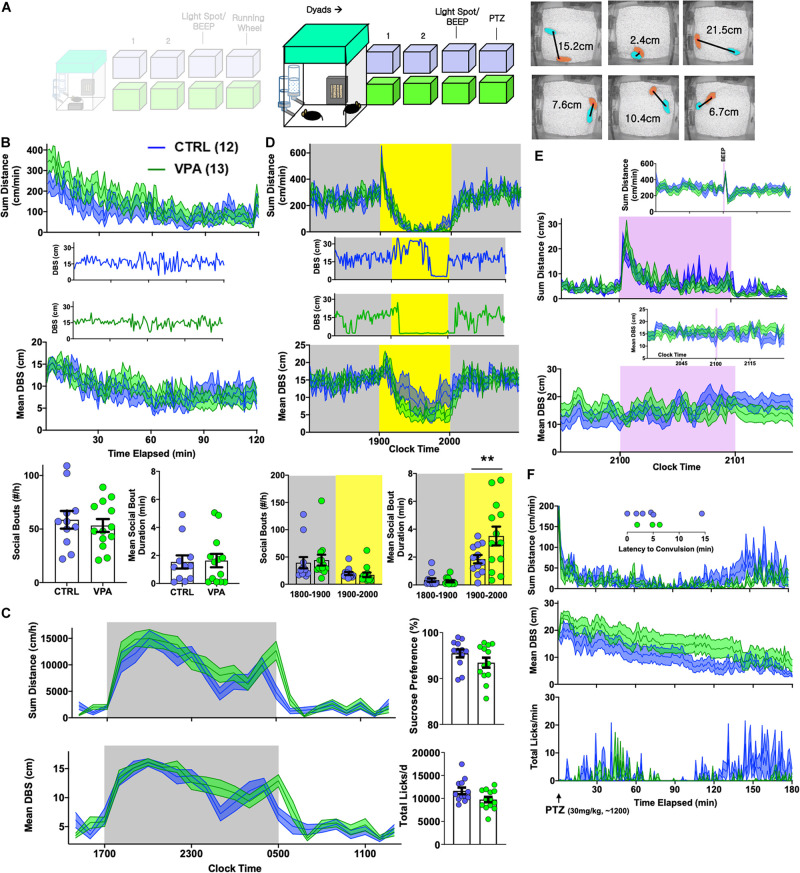
Effects of prenatal VPA exposure on dyadic behavior. **(A)** Sex- and group-matched dyads were paired within BMU home cages [CTRL *n* = 12 pairs (7 female), VPA *n* = 13 pairs (8 female)]. Videotracking was used to calculate proximity (distance between subjects/DBS). **(B)** Continuous measures of total distance and DBS during the initial 2 h of this encounter, with representative examples of DBS. BOTTOM: Total social bouts and mean social bout durations. **(C)** Hourly measures of total distance and mean DBS, with average sucrose preference and total licks (day 2). **(D)** Light spot testing revealed greater social proximity in prenatal VPA-exposed dyads, and results of representative dyads are shown. **(E)** “Beep” test and **(F)** dynamic changes in distance, DBS and licking behavior following a 30 mg/kg PTZ injection applied to both members of the dyad, with inset depicting latency to convulsive seizures. Mean ± SEM shown for all. CTRL, control; VPA, valproic acid. ^∗∗^*p* < 0.01.

## Results

### On the Initiation of Valproic Acid Exposure

VPA is pharmacodynamically complex (inhibiting various ion channels, GABA degradation enzymes, and histone deacetylases) and displays complex pharmacokinetics, with significant protein binding and biologically active metabolites with varied brain clearance rates (Nau and Loscher, 1982; Ghodke-Puranik et al., 2013). As an anticonvulsant, VPA dose requirements vary widely, resulting in poor correlations between plasma concentrations and anticonvulsant efficacy (Nau and Loscher, 1982). Thus, rather than aim for a specific plasma or brain concentration, we began by exploring the effects of VPA dissolved in drinking water (*o.s.*) at a dose range of ∼500–600 mg/kg/d, previously demonstrated to provide some seizure protection in rodent models (Frisch et al., 2009; Smeland et al., 2012; De Caro et al., 2019; Citraro et al., 2020). Eight to nine weeks old C57BL/6J mice were admitted to BMU home-cage chambers fitted with a single lickometered water source (0.8% sucrose drinking water), an infrared-lucent shelter and food hopper (fitted to sense beam breaks). Following two 23 h long baseline recordings, mice were randomized to receive treatment with either control or VPA drinking solutions at intended doses of 200 (days 3,4), 400 (days 5,6) and 600 mg/kg/d (days 7,8, Figure 1A). We hypothesized that behavioral parameters would remain relatively stable in controls, while VPA-treated mice would display dose-dependent changes in activity and/or neurovegetative function. Surprisingly, *both* groups displayed downward drifts in daily total distances accompanied by gradual increases in sheltering and non-invasively computed “sleep” (Figure 1B, see section “Materials and Methods” Jankovic et al., 2019). Stepwise increases in VPA dose were not associated with evidence of acute taste aversion, as assessed by lickometry (Supplementary Figure S1). On day 8, VPA-exposed mice consumed an average actual dose of 616 mg/kg/d (range: 337–959), which *positively* correlated with licks/day (*r* = +0.59, *p* < 0.05) *and* total daily distances (*r* = +0.46, *p* = 0.09, *n* = 14), the latter hinting toward dose-dependent *activation* rather than sedation. Compared with controls, VPA-treated mice displayed a modified average time budget (Jankovic et al., 2019), accumulating greater total shelter time at the cost of feeding. Overall, hourly measures of total distance and sheltering were largely similar between groups (Figures 1C,D).

To resolve finer changes in activity patterns, we visualized daily actograms resolved to cm/min (Figure 1E). In natural environments, animals oscillate between *active* (patrolling, foraging, grooming) and *inactive* states (rest, sleep or quiet wakefulness). In mice, active state structure varies exquisitely by background strain (Hillar et al., 2018) and is modulated by environmental and genetic factors, including alterations in energy balance (Goulding et al., 2008). Unlike states of consciousness (e.g., wakefulness or sleep) that are formally defined electroencephalographically, active states can be discerned through quantitative and continuous measures of behavioral output. We defined active states as at least 1 min long epochs displaying a sustained mean velocity of 5 cm/min (Supplementary Figure S2). While active states in both groups evolved to become more frequent and shorter in duration, higher doses of VPA produced a deficit in total active state time (Figure 1E). Over the last 2 days (at ∼600 mg/kg/d), we applied a series of provocative maneuvers. During a light spot test (Aarts et al., 2015; Jankovic et al., 2019), which imposes a conflict between nocturnal foraging behavior and light avoidance, VPA-treated mice displayed similar patterns of hypoactivity and shelter entry, but prematurely emerged from their shelters toward the stimulus end. An hour later, a 60 s long “beep” produced a transient activity spike and shelter entry that was similar between control and VPA-treated mice (Figure 1F). To obtain a final measure of wellbeing, we affixed running wheels within their home-cages. Compared with controls, VPA-treated mice displayed increased early interest in wheel-running and similarly robust rates of nocturnal running (Figure 1G). Together, these results depict the early behavioral response to *o.s.* VPA therapy, characterized by slight activity reductions without evidence of neophobia, altered sensory processing or diminished exercise motivation.

### On Ethograms of Chronic VPA Exposure

To profile the tolerability of chronic *o.s.* VPA, group-housed 5–6-week old C57BL/6J mice were exposed to either VPA or control solution for 4 weeks within our vivarium (Figure 2A), during which VPA-treated mice consumed approximately 500–700 mg/kg/d without altering weight gain (Figures 2B,C). Mice were then transferred to BMU home-cages fitted with *two* lickometered water sources (water vs. 0.8% sucrose), and VPA exposure was continued (for VPA-treated mice only) in *both* bottles. On day 2, we observed no changes in total daily distances or “sleep,” but VPA-treated mice displayed significantly greater feeding behavior and trended to display lower sucrose preference (*p* = 0.07, Figures 2D–F). Chronic VPA did not impart changes in active state frequency or mean duration, and both groups displayed similar responses to light spot and “beep” provocations (Figures 2G–I). While lowered sucrose preference classically suggests anhedonia, VPA-treated mice displayed a marked increase in wheel-running and contained significantly fewer non-runners (Figure 2J) (Novak et al., 2012) (χ^2^ = 9.62, *p* < 0.01). Finally, all mice received a daytime (∼1,200) intraperitoneal injection of pentylenetetrazole (PTZ). In identical home-cages, we have previously shown that subconvulsant dose PTZ injections in C57BL/6J mice acutely produce immobility with deficits in sheltering. Repeated PTZ injections (kindling) improve latencies to shelter re-entry, albeit at the expense of increased convulsion likelihood (Jankovic et al., 2019). In this light, VPA ingestion produced a behavioral profile resembling *early* PTZ injections, with fewer tonic-clonic convulsions (χ^2^ = 2.39, *p* = 0.1) but more profoundly impaired sheltering (Figure 2K). Thus, at doses that modify behavioral responses to PTZ, chronic *o.s.* VPA produces hyperphagia and increased voluntary wheel-running without significantly altering sensorium, sleep or active state structure.

### On the Early Consequences of Pangestational VPA Exposure

To measure neuropsychiatric teratogenicity, 8–9 weeks old sexually naïve C57BL/6J breeder pairs were assembled and randomized to receive either control solution or VPA. At parturition, bottles were replaced with sucrose-free drinking water (Figure 3A). VPA exposure in this manner prolonged the latency to a viable litter by ∼2 weeks, without impacting litter size (Figure 3B) or sex ratios (control: 57% female, VPA: 48% female, *p* > 0.1). Externally obvious sequelae of neural tube maldevelopment or other structural anomalies were not observed in either group. We next sought to examine how pangestational VPA exposure impacted early neuropsychiatric development without imposing prolonged maternal separation (Tractenberg et al., 2016). Most available tests to assess early murine neurological milestones emphasize sensorimotor reflexes (e.g., cliff avoidance, negative geotaxis) and are subjectively scored on ordinal scales (Heyser, 2004). To achieve unbiased quantitative measurements of pup behavior suitable for automated analysis, we devised a “pup open field” and conducted 15 min long aerial video recordings that were subsequently video-tracked. We first examined a large cohort of unmanipulated C57BL/6J pups assessed between P5 (post-natal day 5) and P15. Total open field distances remained stably low until ∼P12, corresponding to the timing of eye opening (Heyser, 2004). In contrast, open field center times displayed two stepwise decrements at P9 and P12, illustrating the early emergence of thigmotaxis prior to eye opening (Figures 3C,D). Control and VPA-exposed pups behaved similarly at P5 and P10. At P15, VPA-exposed pups displayed significantly lower total open field distances without changes in center time or maximum velocity (Figures 3E,F) without significant sex-related differences (Supplementary Figures S3A,B). Heat maps of position probability identified large individual variations in field exploration across both groups (Figure 3G). VPA-exposed pups were less likely to explore all four corners of the open field (χ^2^ = 9.62, *p* < 0.05), collectively illustrating a deficit in early measures of spontaneous exploratory behavior.

### On the Effects of Pangestational VPA Exposure in Adulthood

When first introduced to BMU home-cages at ∼8–9 weeks of age, prenatal-VPA exposed adult mice displayed similar patterns of locomotor habituation with slightly greater total sheltering times (*p* = 0.1, Figure 4B). On their second baseline day, VPA-exposed mice displayed similar rates of overall movement, sleep and sucrose preference. Daily time budgets revealed a relative increase in sheltering behavior at the cost of feeding and “other” activities (time spent *not* feeding, drinking or sheltering, Figures 4C,D). VPA-exposed mice displayed a significantly greater number of daily active states (∼81 vs. 71/day in controls) that were of significantly shorter duration (∼10.7 vs. 9 min), and a reduced total active state time (Figure 4E). Across these variables, no significant sex-based differences were observed (Supplementary Figures S3C,D and Supplementary Table S1), with the exception of overall “sleep” which was significantly lower in female mice (Paul et al., 2006). Light spot and “beep” tests revealed no significant group differences (Figure 4F). When presented with a running wheel, VPA-exposed mice displayed significantly greater average rates of nocturnal wheel running (Figure 4G).

Autistic endophenotypes of the human fetal VPA syndrome (Bromley et al., 2014; Velez-Ruiz and Meador, 2015; Bromley and Baker, 2017; Veroniki et al., 2017) have been ascertained in rodent prenatal VPA protocols using tasks of sociability, social novelty and/or ultrasonic vocalizations (Kazdoba et al., 2016; Nicolini and Fahnestock, 2018). To examine how prenatal VPA exposure altered the dynamics of social behavior within a now familiar home-cage setting, we next paired sex- and drug exposure-matched mice into “dyads” (CTRL-CTRL vs. VPA-VPA, Figure 5A). Rather than manually score specific social behaviors like “sniffing” or “following” (Crawley, 2007), we simultaneously tracked both mice to capture a continuous readout of inter-mouse proximity, termed DBS (distance between subjects). In the first 2 h, mean DBS levels reduced from ∼15 to 10 cm in both groups, paralleling a locomotor habituation response (Figure 5B). We then closely examined the morphology of individual social bouts, which we defined as epochs lasting ≥ 3 s where DBS was consistently ≤ 4 cm. Prenatal VPA exposure did not impact the frequency or the mean duration of social bouts (Figure 5B). Over the subsequent 2 days, circadian alterations in mean proximity generally mirrored locomotor fluctuations (Peleh et al., 2020). Prenatal VPA-exposed dyads displayed a delayed behavioral anticipation of the light phase, but were nevertheless noted to rest together during the light phase (Figure 5C). Social bouts were generally brief during the dark phase, but during a light spot challenge, VPA-exposed mice displayed significantly longer social bout durations (Figure 5D). “Beep” testing did not elicit differences in dyadic responses (Figure 5E). Finally, to interrogate seizure threshold, we applied subconvulsant dose PTZ injections to all mice. While convulsions occurred at a low rate in both groups (χ^2^ = 0.75, *p* > 0.3), home-cage measures of PTZ response were distinct: VPA-exposed mice displayed a blunted motor recovery and remained more distant from their counterparts (Figure 5F), suggesting enhanced seizure severity (Choi et al., 2016).

## Discussion

In the 1960s, after the serendipitous discovery of VPA’s anticonvulsant actions, early clinical trials with VPA were conducted in psychiatric asylums, often enriched with epileptic patients. Valproate displayed anticonvulsant *and* beneficial psychotropic properties, with improvements noted in depression, “viscosity” and occasionally mild euphoria (Harris et al., 2003; Lopez-Munoz et al., 2018). Following its release in the United States in 1978, VPA experienced enormous popularity both as a broad-spectrum anticonvulsant and mood stabilizer, and our knowledge of VPA’s systemic adverse effects expanded, including pancreatic/hepatic toxicity, platelet dysfunction, weight gain, sexual dysfunction (Verrotti et al., 2016) and tremor (Nanau and Neuman, 2013). A thorough understanding of the structural and cognitive consequences of fetal VPA exposure did not emerge until the 1990s–2000s, a rather extended and potentially preventable delay that contributed to significant adverse developmental outcomes (Meador and Loring, 2016). Rodent studies on VPA’s psychopharmacological effects lagged well behind human clinical experience, with demonstrations of anxiolytic-like effects (Shephard et al., 1985), antidepressant-like effects (Fernandez Teruel et al., 1988), and evidence of “autism-like” behavior in rodents prenatally exposed to VPA (Schneider and Przewlocki, 2005).

Today’s newest generation of anticonvulsants are intelligently designed through drug discovery programs that combine the latest technologies in high-throughput screening and medicinal chemistry with an array of established anticonvulsant assays (Rogawski and Hanada, 2013; Klitgaard et al., 2016; Wilcox et al., 2020). Still, evaluations of tolerability crudely focus on motor *toxicity*, which itself poorly correlate with patient complaints of treatment-related somnolence or fatigue (Mead et al., 2016). Similarly, animal studies on teratogenicity focus primarily on structural malformations, and standardized assessments of neurodevelopmental behavioral toxicity have been consistently deferred and/or ignored. In this study, we asked whether detailed automated evaluations of home-cage behavior would be sensitive enough to detect anticonvulsant psychotropic effects, and further, reveal features of pervasive neurodevelopment (if any) in mice exposed to that anticonvulsant prenatally. We chose VPA as a prototypical AED known to display both such features, and employed C57BL/6J mice as subjects, given their widely reproducible home-cage ethograms (Loos et al., 2014; Robinson et al., 2018; Jankovic et al., 2019) and comparatively low rates of within-strain variability (Loos et al., 2015). Home-cage monitoring is grounded on the principle that *if* a rodent were to experience clinically meaningful symptoms of autism, psychomotor delay, anxiety or depression, such a syndrome would exert measurable effects on activities of daily living as measured in *their* home-cages. Extended recording durations highlight phenotypes which may be only evident during a specific circadian phase. This is commonly observed in actigraphic evaluations of patients with psychiatric disorders. As an example, the *psychomotor retardation* of major depression is primarily a daytime phenomenon (Volkers et al., 2002). In view of this point, home-cage monitoring is unparalleled in its ability to characterize the complex behavioral features of the murine “night.”

We devised two paradigms to assess the tolerability of VPA ingestion. First, we examined how mice responded to the initial introduction of VPA during a gradual dose uptitration schedule. In daily recordings conducted over 8 days, control mice displayed downward drifts in overall cage exploration, with gradual increases in sheltering, feeding and “sleep.” Active states also evolved to become more frequent and shorter in duration in control mice. These findings may relate to infradian rhythms and/or represent an *observer* effect: by *individually* housing our mice (albeit within an enriched home-cage) to *individually* assess changes in behavior, we observed phenotypic drifts that may be secondary to the cumulative effects of social isolation. Among various trajectories measured, VPA at doses of 400–600 mg/kg/d diminished total active state time without significantly altering the mean duration of active states. We infer that VPA, gradually introduced in this manner, made mice somewhat “less active.” Nevertheless, VPA consumption did not alter voluntary wheel-running behavior, a task that is broadly sensitive to deficits in motivation, neuromuscular function and/or neophobia (Novak et al., 2012).

In the second approach, we applied similar doses of VPA chronically to group-housed mice. While we did not observe changes in weight gain over the treatment period, VPA-treated mice accumulated significantly greater feeding durations and entries. Weight gain is a well-known side effect of VPA and may relate to increased appetite, although appetite (or satiety/hunger) are challenging quantities to objectively measure in humans (Gibbons et al., 2019). VPA has been linked to hyperinsulinemia (and insulin resistance), and hyperleptinemia (with leptin resistance), both of which would be expected to increase appetite (Greco et al., 2005; Nanau and Neuman, 2013). A mild reduction in sucrose preference was observed, consistent with lower natural reward sensitivity (*anhedonia*). Nevertheless, VPA-treated mice displayed a robust increase in overall wheel-running. This may be interpreted as adaptive and beneficial (antidepressant-like, pro-resilient) or maladaptive and pathological (pro-manic, “compulsive/addiction-prone”) (Novak et al., 2012). Operant tasks that reward lever-pressing with wheel access, or more invasive measures of motivational states (such as intracranial self-stimulation) may help to resolve these discrepant interpretations. Fundamentally, we conclude that these data emblemize VPA’s effects on the distributed neural circuits underlying reward and energy balance, shifting the setpoint toward increased energy consumption *and* expenditure.

We were surprised to observe that at a dose range deemed to be generally “tolerable,” VPA-exposed breeder pairs required at least 2 weeks longer to produce a litter of pups. Multiple mutually non-exclusive mechanisms relating to male and female factor may explain this subfertility (Verrotti et al., 2016), including endocrine imbalances that impact gonadal health as well as reductions in libido/sexual drive, that may be congruent with deficits in sucrose preference. VPA-exposed pups were generally normal in appearance and by P15 (but not earlier), displayed reduced exploratory behavior on our pup open field assay, without overt evidence of frank neurological motor impairment. As adults, hourly or total daily distances were unchanged by prenatal VPA exposure. However, VPA-exposed mice displayed more frequent active states that were significantly shorter in duration, illustrating a fundamental derangement in the temporal organization of activity, and phenocopying the effects of social isolation (Figure 1). Such abnormalities in the rhythms of daily activity may be comorbid with distinct VPA-induced circadian abnormalities (Tsujino et al., 2007): these parameters (including *tau* or period length) were not specifically measured in our study using classical free-running constant-darkness conditions (Eckel-Mahan and Sassone-Corsi, 2015). In conjunction with changes in active state morphology, VPA-exposed mice also displayed an increase in sheltering at the cost of feeding time, a theme (or “trait” or “mood”) that we have previously identified in mice modeling Dravet Syndrome *and* in mice subjected to daily PTZ-induced seizures (Jankovic et al., 2019). Having access to a running wheel was sufficient to overcome their proclivity to shelter, with VPA-exposed mice displaying significantly greater rates of nocturnal wheel-running. In the context their presumed “autism-like” phenotype, we conjecture that increased wheel-running may relate to a tendency to engage in repetitive stereotyped behaviors. Finally, while a wealth of studies have identified sex-based differences in autistic or autism-like phenotypes following prenatal VPA exposure across humans and rodents (Schneider et al., 2008; Christensen et al., 2013; Kim et al., 2013; Melancia et al., 2018; Kazlauskas et al., 2019; Scheggi et al., 2020), we did not observe exaggerated phenotypes in male mice (Supplementary Figure S3). This discrepancy may relate to our particular mode of VPA administration (i.e., dissolved in drinking water), and/or our ethologically centered approach to assessing such endpoints almost exclusively within the home-cage, thereby minimizing olfactory cues that may alter stress-sensitive measures of emotionality (Sorge et al., 2014).

Reductions in social interest, assayed through measures of social play in rats or social exploration in mice, have made the prenatal VPA protocol a widely popular model of autism (Roullet et al., 2013; Nicolini and Fahnestock, 2018). The vast majority of these efforts have employed out-of-cage assessments of sociability, such as the three-chamber task (Kazdoba et al., 2016). Recently, more continuous home-cage based assessments of social behavior have employed implanted radio-frequency identification (RFID) chips (Peleh et al., 2020). In our experiments, we applied video-tracking to study dyadic behavior. With continuous, automated and unbiased measures of proximity, we observed no evidence of social withdrawal during their initial interaction. Further, circadian variations in mean proximity were largely similar (Peleh et al., 2020). During the light spot test (Aarts et al., 2015; Jankovic et al., 2019), VPA-exposed dyads displayed more prolonged social bouts, revealing if anything, a tendency to *seek* a social partner during an aversive or stressful stimulus. Together, these results call into question the validity of short, subjectively scored assays of social exploration and suggest that broad inferences about “impaired sociability” (Krishnan et al., 2017) may not necessarily translate to truly pervasive changes in social behavior, at least within dyads.

We concede two main limitations to this work. First, by applying VPA dissolved in drinking water, our intention was to avoid the stress of daily intraperitoneal injections, which are also not without risk when applied to a gravid mouse. Day time reductions in fluid intake (Jankovic et al., 2019) may have resulted in low day time VPA plasma/serum levels (Loscher and Nau, 1982; Frisch et al., 2009). VPA’s “carry-over” anticonvulsant effects during a daytime PTZ challenge may be explained by more pervasive increases in brain GABA content, which have been previously shown to more accurately correlate with VPA-induced increases in convulsive threshold (Loscher and Nau, 1982; Nau and Loscher, 1982). Mini-osmotic pumps (delivering VPA cerebroventricularly) or subcutaneously absorbed pellets would have been alternative approaches to provide continuous VPA exposure selectively to the female breeder. However, these are invasive options that may themselves impede mating and are not straightforward to terminate (e.g., at parturition). Further, by escaping first pass metabolism, these alternate dosing strategies may result in an altered pattern of metabolites, many of which are known to be biologically active and subserve anticonvulsant roles (Loscher and Nau, 1982; Nau and Loscher, 1982; Ghodke-Puranik et al., 2013; Verrotti et al., 2016).

Second, to maximize translational potential, epileptic mice (with spontaneous seizures) would have made for more ideal subjects. These are not a unique entity: many epileptic mouse models are currently available and may each have unique home-cage behavioral signatures. For example, mice with a Dravet syndrome mutation display nocturnal hypoactivity and increased sleep (Jankovic et al., 2019), while nocturnal *hype*ractivity is observed in mice modeling temporal lobe epilepsy (Wulsin et al., 2018). Ideally, to examine how behavioral side effects correlate with anti-seizure efficacy, such studies would be performed with simultaneous electroencephalographic (EEG) monitoring, which may itself impose changes in cage exploration and neurovegetative function. Therefore, as a first pass, we examined VPA’s effects in non-epileptic mice. With improvements in the ergonomics of wireless EEG, we envision a future where preclinical assessments of tolerability will be conducted in an individualized fashion, at doses that are carefully titrated to the cessation of spontaneous seizures.

## Conclusion

In conclusion, this work presents a novel paradigm to assess psychotropic and cognitive/behavioral teratogenic side effects of AEDs in mice. Our approach may be extended to any candidate/pipeline neurotherapeutic that would necessitate daily intake and may guide initial safety recommendations regarding ingestion during pregnancy for agents with limited human safety data.

## Data Availability Statement

The raw data supporting the conclusions of this article will be made available by the authors, without undue reservation, to any qualified researcher.

## Ethics Statement

The animal study was reviewed and approved by the Institutional Animal Care and Use Committee at the Baylor College of Medicine.

## Author Contributions

VK conceived and designed the study and drafted the manuscript. JB and VK designed the figures. JB, AT, LT, MJ, PK, and CS contributed to data acquisition and analysis. All authors contributed to the article and approved the submitted version.

## Conflict of Interest

VK receives additional funding from SK Life Science Inc. for contract laboratory research that is unrelated to the topic of this manuscript. VK is a member of the Epilepsy Research Benchmark Stewards Committee (American Epilepsy Society/National Institutes of Neurological Disorders and Stroke). The remaining authors declare that the research was conducted in the absence of any commercial or financial relationships that could be construed as a potential conflict of interest.
