# Supplementary material for: On the Digital Psychopharmacology of Valproic Acid in Mice
Source: Front Neurosci. 2020 Nov 6;14:594612. doi: 10.3389/fnins.2020.594612 (PMC7677503; doi:10.3389/fnins.2020.594612)
Supplement: Supplementary file 1 [file Data_Sheet_1.PDF]

Supplementary Material

Figure S1

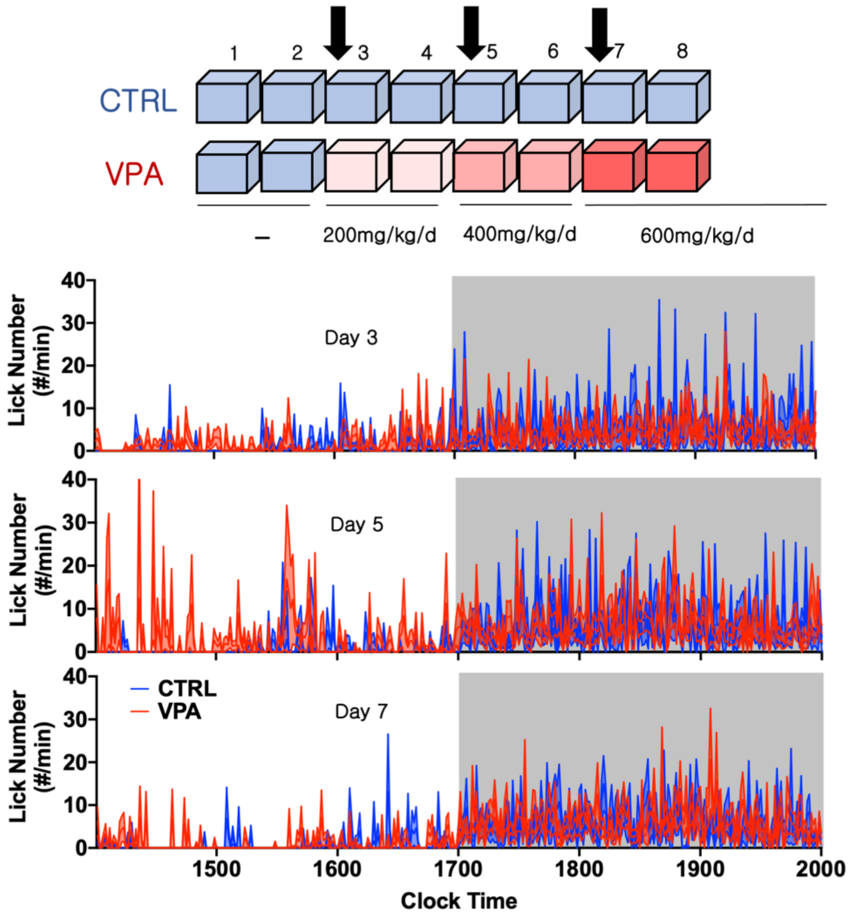

Supplementary Figure 1. In Fig.1, drinking solutions were replaced for all mice between 1300-1400 on days 3, 5 and 7. CTRL mice received a fresh solution of 0.8% sucrose-drinking water, while VPA-treated mice received fresh solutions with increasing concentrations of VPA. Stepwise increases in VPA concentration did not produce an obvious impact on licking behavior. Mean ± S.E.M shown for all. CTRL: control, VPA: valproic acid.

Figure S2

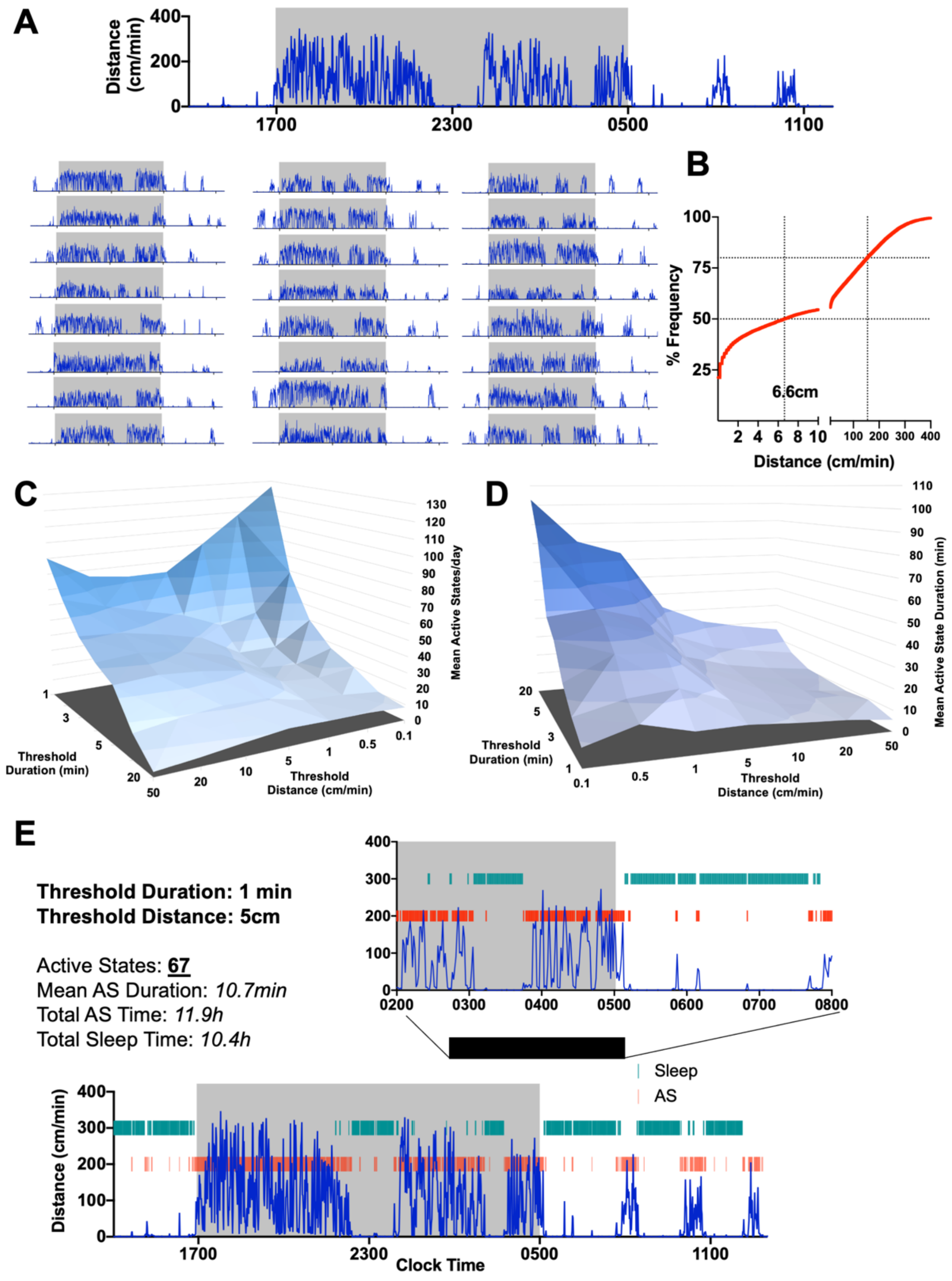

Supplementary Figure 2: Active state thresholds. (A) Actograms for all C57BL/6J mice [n=25, 12 female] from day 2 (Fig. 1). (B) Cumulative distribution histogram of minutely distance measures, ranging from 0-400cm/min, with 50th percentile corresponding to ~6.6cm. (C) 3-dimensional graph depicting how changes in threshold duration and distance modulate total daily active state numbers, and (D) mean active state duration. Larger duration and distance thresholds effectively ignore short active states. (E) Depiction of active states (AS, red) for a representative mouse at a threshold duration and distance of 1 minute and 5cm/min respectively. Epochs of behaviorally defined "sleep" are shown in green.

Figure S3

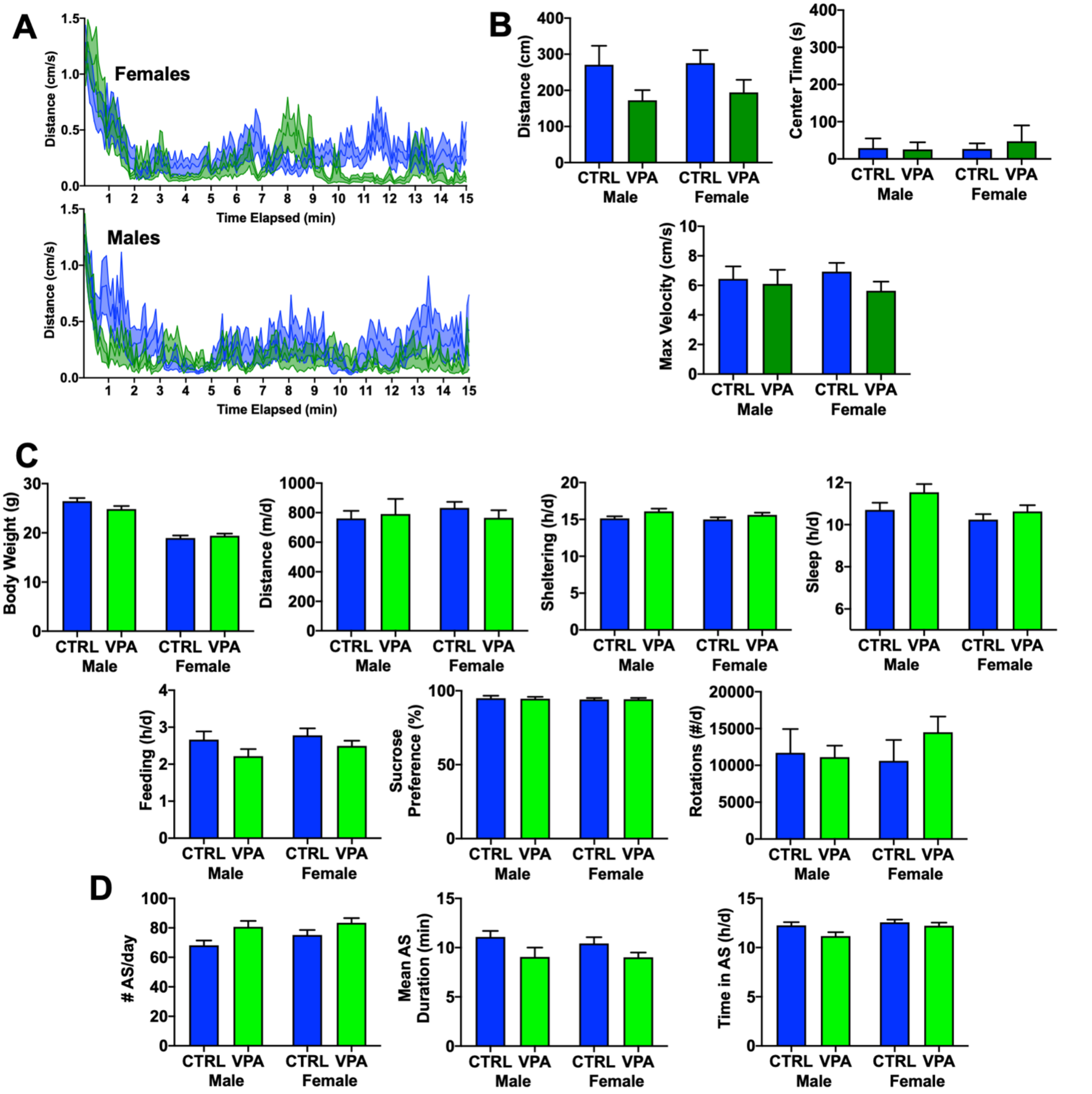

Supplementary Figure 3: Sex Differences following prenatal VPA exposure. (A) Pup open field locomotor habituation at P15 (corresponding to Fig. 3F) separated by male and female mice. (B) Total distances, center times and maximum velocities for P15 pups. (C) Sex-based differences in adult home-cage measures (from Fig. 4) and (D) active state morphology following prenatal VPA exposure. Mean $\pm$ SEM shown for all. CTRL: control, VPA: valproic acid.

Table S1

Figure	Fixed effects (type III)	P value	P value summary	F (DFn, DFd)
1B (Distance)	Day (3-8)	<0.0001	****	F (2.462, 56.62) = 10.16
	Treatment	0.5684	ns	F (1, 23) = 0.3349
	Day x Treatment	0.9149	ns	F (5, 115) = 0.2949
1B (Sleep)	Day (3-8)	<0.0001	****	F (2.829, 65.08) = 10.76
	Treatment	0.3266	ns	F (1, 23) = 1.005
	Day x Treatment	0.5287	ns	F (5, 115) = 0.8332
1B (Sheltering)	Day (3-8)	0.0004	***	F (3.207, 73.76) = 6.615
	Treatment	0.9556	ns	F (1, 23) = 0.003164
	Day x Treatment	0.6122	ns	F (5, 115) = 0.7167
1B (Feeding Duration)	Day (3-8)	0.3567	ns	F (3.437, 80.42) = 1.104
	Treatment	0.9418	ns	F (1, 24) = 0.005446
	Day x Treatment	0.071	ns	F (5, 117) = 2.094
1B (Lick Duration)	Day (3-8)	0.116	ns	F (2.998, 67.16) = 2.044
	Treatment	0.2358	ns	F (1, 23) = 1.482
	Day x Treatment	0.1152	ns	F (5, 112) = 1.817
1D (Distance)	Time (h)	<0.0001	****	F (7.461, 171.6) = 48.01
	Treatment	0.5886	ns	F (1, 23) = 0.3009
	Time x Treatment	0.8401	ns	F (22, 506) = 0.7013
1D (Sheltering)	Time (h)	<0.0001	****	F (7.710, 177.3) = 44.02
	Treatment	0.3136	ns	F (1, 23) = 1.061
	Time x Treatment	0.5058	ns	F (22, 506) = 0.9668
1E (Active State Number)	Day (3-8)	0.0007	***	F (3.127, 71.91) = 6.238
	Treatment	0.1177	ns	F (1, 23) = 2.641
	Day x Treatment	0.5268	ns	F (5, 115) = 0.8359
1E (Active State Duration)	Day (3-8)	<0.0001	****	F (2.485, 57.15) = 9.916
	Treatment	0.8599	ns	F (1, 23) = 0.03187
	Day x Treatment	0.4176	ns	F (5, 115) = 1.006
1E (Total AS Time)	Day (3-8)	<0.0001	****	F (3.682, 84.68) = 12.62
	Treatment	0.1531	ns	F (1, 23) = 2.183
	Day x Treatment	0.1009	ns	F (5, 115) = 1.893
1G (Wheel Rotations)	Time (h)	<0.0001	****	F (4.657, 102.5) = 20.23
	Treatment	0.5632	ns	F (1, 22) = 0.3445
	Time x Treatment	0.6411	ns	F (21, 462) = 0.8622
2C (Body Weight)	Week	<0.0001	****	F (1, 39) = 80.91
	Treatment	0.1631	ns	F (1, 39) = 2.021
	Week x Treatment	0.5696	ns	F (1, 39) = 0.3289
2D (Distance)	Time (h)	<0.0001	****	F (8.689, 338.9) = 70.11
	Treatment	0.9807	ns	F (1, 39) = 0.0005927
	Time x Treatment	0.0068	**	F (22, 858) = 1.920
2D (Sheltering)	Time (h)	<0.0001	****	F (8.655, 337.5) = 81.12
	Treatment	0.1107	ns	F (1, 39) = 2.664
	Time x Treatment	0.3069	ns	F (22, 858) = 1.130
2D (Feeding Entries)	Time (h)	<0.0001	****	F (6.161, 240.3) = 41.46
	Treatment	0.172	ns	F (1, 39) = 1.936
	Time x Treatment	0.0309	*	F (22, 858) = 1.648
2J (Wheel Rotations)	Time (h)	<0.0001	****	F (3.648, 138.6) = 16.60
	Treatment	0.0027	**	F (1, 38) = 10.34
	Time x Treatment	<0.0001	****	F (20, 760) = 3.569
4C (Distance)	Time (h)	<0.0001	****	F (9.459, 539.2) = 74.80
	Treatment	0.7746	ns	F (1, 57) = 0.08279
	Time x Treatment	0.3861	ns	F (22, 1254) = 1.060
4C (Sheltering)	Time (h)	<0.0001	****	F (11.17, 636.4) = 83.41
	Treatment	0.0141	*	F (1, 57) = 6.417
	Time x Treatment	0.2649	ns	F (22, 1254) = 1.171

Figure	Fixed effects (type III)	P value	P value summary	F (DFn, DFd)
4C (Feeding Entries)	Time (h)	<0.0001	****	F (8.606, 481.9) = 59.05
	Treatment	0.1576	ns	F (1, 56) = 2.052
	Time x Treatment	0.0386	*	F (22, 1232) = 1.601
4G (Wheel Rotations)	Time (h)	<0.0001	****	F (5.097, 262.8) = 30.89
	Treatment	0.0476	*	F (1, 53) = 4.113
	Time x Treatment	0.0022	**	F (20, 1031) = 2.164
5C (Sum Distance)	Time (h)	<0.0001	****	F (7.490, 172.3) = 46.30
	Treatment	0.2372	ns	F (1, 23) = 1.473
	Time x Treatment	0.0063	**	F (22, 506) = 1.948
5C (Distance Between Subjects)	Time (h)	<0.0001	****	F (9.766, 222.8) = 39.40
	Treatment	0.2485	ns	F (1, 23) = 1.402
	Time x Treatment	0.0072	**	F (22, 502) = 1.926
S3B Total Distance (cm) (pups)	Treatment	0.0249	*	F (1, 96) = 5.193
	Sex	0.7391	ns	F (1, 96) = 0.1116
	Treatment x Sex	0.8281	ns	F (1, 96) = 0.04743
S3B Maximum Velocity (cm/s) (pups)	Treatment	0.2902	ns	F (1, 96) = 1.131
	Sex	0.9896	ns	F (1, 96) = 0.0001716
	Treatment x Sex	0.5396	ns	F (1, 96) = 0.3791
S3B Center Time (s) (pups)	Treatment	0.7439	ns	F (1, 96) = 0.1073
	Sex	0.7025	ns	F (1, 96) = 0.1468
	Treatment x Sex	0.636	ns	F (1, 96) = 0.2255
S3C Body Weight (g)	Treatment	0.3319	ns	F (1, 55) = 0.9584
	Sex	<0.0001	****	F (1, 55) = 125.0
	Treatment x Sex	0.0792	ns	F (1, 55) = 3.198
S3C Total Distance (m/d)	Treatment	0.8033	ns	F (1, 55) = 0.06262
	Sex	0.7595	ns	F (1, 55) = 0.09467
	Treatment x Sex	0.5109	ns	F (1, 55) = 0.4379
S3C Sheltering (h/d)	Treatment	0.0206	*	F (1, 55) = 5.680
	Sex	0.3485	ns	F (1, 55) = 0.8943
	Treatment x Sex	0.6202	ns	F (1, 55) = 0.2484
S3C Total Sleep (h/d)	Treatment	0.0796	ns	F (1, 55) = 3.191
	Sex	0.0496	*	F (1, 55) = 4.030
	Treatment x Sex	0.5144	ns	F (1, 55) = 0.4306
S3C Feeding (h/d)	Treatment	0.0604	ns	F (1, 54) = 3.679
	Sex	0.3087	ns	F (1, 54) = 1.056
	Treatment x Sex	0.6789	ns	F (1, 54) = 0.1733
S3C Sucrose Preference (%)	Treatment	0.937	ns	F (1, 55) = 0.006302
	Sex	0.643	ns	F (1, 55) = 0.2172
	Treatment x Sex	0.8518	ns	F (1, 55) = 0.03521
S3C Rotations/d	Treatment	0.5113	ns	F (1, 40) = 0.4392
	Sex	0.6476	ns	F (1, 40) = 0.2121
	Treatment x Sex	0.371	ns	F (1, 40) = 0.8186
S3D Mean AS Duration (min)	Treatment	0.0252	*	F (1, 56) = 5.288
	Sex	0.6409	ns	F (1, 56) = 0.2200
	Treatment x Sex	0.6759	ns	F (1, 56) = 0.1766
S3D # of Active States/d	Treatment	0.0061	**	F (1, 56) = 8.122
	Sex	0.1919	ns	F (1, 56) = 1.745
	Treatment x Sex	0.5732	ns	F (1, 56) = 0.3212
S3D Time in AS (h/d)	Treatment	0.0452	*	F (1, 56) = 4.197
	Sex	0.0553	ns	F (1, 56) = 3.832
	Treatment x Sex	0.2943	ns	F (1, 56) = 1.121
